# Fluorescence resonance energy transfer detection methods: Sensitized emission and acceptor bleaching

**DOI:** 10.3892/etm.2014.1928

**Published:** 2014-08-22

**Authors:** JIE QIAN, BINGBO YAO, CHUANYUE WU

**Affiliations:** 1 School of Life Science and Technology, Tongji University, Shanghai 200092, P.R. China; 2 South University of Science and Technology of China, Shenzhen, Guangdong 518055, P.R. China

**Keywords:** protein-protein interaction, fluorescence resonance energy transfer, sensitized emission, acceptor bleaching

## Abstract

The present study compared the advantages and disadvantages of fluorescence resonance energy transfer (FRET) determination technologies, namely, sensitized emission (SE) and acceptor bleaching (AB), in order to analyze the applicability of SE and AB for studies investigating particularly interesting new cysteine histidine-rich protein 1 (PINCH1)/integrin-linked kinase (ILK) interaction. HeLa cells were transfected with cyan fluorescent protein (CFP)-PINCH1 and yellow fluorescent protein (YFP)-ILK to establish a PINCH1/ILK interaction examination model. PINCH1/ILK interactions in different parts of the cells were also examined by SE and AB. The FRET determination technologies SE and AB were able to examine PINCH1/ILK interaction. SE was more sensitive for FRET determination and thus had greater reliability. Therefore, SE is highly commended for membrane protein-protein interaction studies.

## Introduction

Fluorescence resonance energy transfer (FRET) detection methods are able to detect living cells and interactions between proteins in fixed cells. FRET has been widely used for real-time detection of the activation process of enzymes in apoptosis (1) and for studying the function of proteins in the process of transcription (2) and the activation of gene transcription (3,4). The method has also been applied in drug screening (5), the formation of compounds (6) and other fields of research. FRET detection methods include sensitized emission (SE) and acceptor bleaching (AB), as well as fluorescence lifetime imaging microscopy and spectroscopy (7,8). Based on the SE detection approach, a previous study (9) has revealed that parathyroid hormone (PTH) is able to generate changes in FRET through protein kinase C (PKC)-activated reporter molecules. These studies have confirmed the role of the PKC-activated reporter molecule in PKC-related signal transduction, providing a new platform for the study of the signal transduction of PTH. Aside from its use in studies investigating the interactions between proteins, the AB detection approach has also been used to examine the E protein domain at the spindle checkpoint associated with the Mps1 protein (10) and to study the formation of β-secretase dimer (11).

To be successfully implemented, FRET has three essential factors: i) the emission spectrum of the donor must overlap with the excitation spectrum of the acceptor; ii) the distance between the donor and the acceptor must be sufficiently close and; iii) the dipoles of the donor and the acceptor must have a certain spatial orientation (12,13).

When the donor and the acceptor are sufficiently close together, fluorophores, in their excited state, transfer energy to the adjacent acceptor molecules in a non-radiative manner, through interaction between dipoles (14,15).

The FRET effect is proportional to the sixth power of the distance between molecules, as shown in Formula 1 (16):

Formula 1 $$\text{E}=\frac{1}{1+{\left(\frac{\text{r}}{{\text{R}}_{0}}\right)}^{6}}$$

where E is the FRET effect; r is the distance between fluorophores and; R_0_ is the distance between the donor and acceptor fluorophores when the energy transfer effect is 50%. Different FRETs have different R_0_ values, where R_0_ refers to the fluorescence eigenvalue; a different fluorescence has different R_0_ values that may be calculated based on its characteristics.

Classic FRET detection methods include SE and AB. Given the photophysical properties of organic fluorophores, the most current FRET pairs exhibit the spectral bleed-through phenomenon. The impact of spectral crosstalk must be eliminated in the quantitative detection of the FRET effect. In FRET detection, the SE method is used to eliminate the impact of spectral crosstalk, differences in fluorescent protein expression, background noise and other factors by establishing control groups and calculating correction factors.

When detecting the sample, the images of the donor, FRET and acceptor channels are collected simultaneously. The net density of the region of interest (ROI) is evaluated, and is included in the SE calculation (Formula 2) so that the FRET efficiency value may be obtained (1).

Formula 2 $$\text{E}=\frac{\text{B}-\text{A}\times \beta -\text{C}\times (\gamma -\alpha \times \beta )}{\text{C}\times (1-\beta \times \delta )}$$

In Formula 2, A is the bare density of the selected area in the images captured of the donor channel (bare density = protein fluorescence density - background noise); B is the bare density of the selected area in the images captured of the FRET channel; C is the bare density of the selected area in the images captured of the acceptor channel; and α, γ, β and δ are the correction factors. Among these, β may only be obtained when a donor exists and β=B/A; α, γ and δ may only be obtained when the acceptor exists and α=A/C, γ=B/C and δ=A/B.

Based on the FRET principle, when the photon generation efficiency of the receptor fluorescence protein decreases, the receptor no longer receives emitted light generated by the donor being activated in order to activate itself to generate a photon. Therefore, the photon generation efficiency of the donor fluorescence protein increases; that is, the fluorescence signal of the donor channel is enhanced. The AB calculation formula (Formula 3) is used to calculate the enhancement of the donor fluorescence and the FRET efficiency prior to the acceptor fluorescent bleaching being obtained, which is the AB method (8).

Formula 3 $$\text{E}=1-\frac{{\text{I}}_{\text{D}}^{\text{b}}}{{\text{I}}_{\text{D}}^{\text{a}}}$$

In Formula 3, I^b^_D_ and I^a^_D_ refer to the fluorescence intensity of the donor prior to and following the fluorescent bleaching, respectively.

In the present study, particularly interesting new cysteine histidine-rich protein 1 (PINCH1) and integrin-linked kinase (ILK) labeled with cyan fluorescent protein (CFP) and yellow fluorescent protein (YFP) fluorophores, respectively, were used to establish a FRET detection model to analyze the interaction between PINCH1 and ILK. The classic FRET detection methods, SE and AB, were used to detect the interaction between PINCH1 and ILK, compare the advantages and the disadvantages of SE with those of AB, and discuss the applicability of SE and AB for membrane protein-protein interaction studies.

## Materials and methods

### Materials and instruments

The HeLa cells used in the present experiment were preserved in the authors’ laboratory. Fetal calf serum was purchased from Hangzhou Sijiqing Biological Engineering Materials Co. (Hangzhou, China). RPMI-1640 medium was obtained from Biowest (Nuaillé, France). The plasmids used were CFP-PINCH1 and YFP-ILK, which were donated by Professor Wu Chuanyue from the University of Pittsburgh (Pittsburgh, PA, USA) and were amplified in the authors’ laboratory. The plasmid amplification kit was purchased from Qiagen (Hilden, Germany). The plasmid transfection kit Lipofectamine^®^ 2000 was obtained from Invitrogen Life Technologies (Carlsbad, CA, USA). Opti-MEM^®^ was purchased from Gibco^®^ (Invitrogen Life Technologies). The confocal microscope and FRET test set were provided by the Tongji University Public Experimental Platform (Leica TCS SP2; Leica Microsystems CMS GmbH, Mannheim, Germany; donor-selected excited light, 458 nm; receptor-selected excited light, 514 nm).

### Cell transfection

Cell transfection was optimized according to the instructions provided by the manufacturers of the Lipofectamine^®^ 2000 transfection reagent.

A total of ~6×10^4^ HeLa cells were placed in each well of a 12-well plate to be cultured overnight. Prior to transfection, the medium in each well was replaced with 400 μl serum-free RPMI-1640 medium. A certain quantity of the transfection reagent A total of 1 μl Lipofectamine^®^ 2000 was placed in a 1.5-ml EP tube. A total of ~100 μl/well Opti-MEM^®^ was added to the solution, which was marked as solution A and left to stand for 5 min. A total of 1 μg CFP-PINCH-1 and 1 μg YFP ILK plasmids were added separately or together into a 1.5-ml EP tube, combined with 100 μl/well Opti-MEM^®^ to produce a solution and subsequently marked as solution B. Solution A was mixed with B briefly and left to stand for 20 min. Mixed A and B solution was added to each well to establish the standing culture. After 4 h, the medium was replaced by a medium containing 10% fetal calf serum (FBS).

### FRET detection methods

#### SE detection method

In the SE method, the images of the donor, FRET and receptor channels when only the donor or receptor existed were collected prior to testing the sample. The bare density of the ROI was determined to obtain the relative correction parameters in Formula 2.

When detecting the co-transfected CFP-PINCH1 and YFP-ILK samples, the images in the CFP, FRET and YFP channels were collected at the same time as the selection of the ROI. Net fluorescence density values of A (CFP channel), B (FRET channel) and C (YFP channel) were obtained by the subtraction of the background light density from the fluorescence density of the protein signal.

The Leica confocal computer software (Leica Microsystems CMS GmbH, Mannheim, Germany) was used to calculate the FRET efficiency at each point in the images in a point-to-point manner based on Formula 2. Thus, the FRET efficiency distribution chart in full view was obtained.

#### AB detection method

In the AB method, the images in the CFP and YFP channels were collected prior to and following receptor fluorescent bleaching. The fluorescent bleaching time period of the receptor depended on the non-reduction of the receptor following fluorescent bleaching. It is possible to repeat receptor bleaching a few times; however, too much bleaching is not recommended. The longer the interval, the higher the possibility of a change in cell state, which adversely affects the experimental results.

To obtain the statistical values of the optical densities for the CFP and YFP channel images of the captured samples prior to and following acceptor fluorescent bleaching, the FRET efficiency distribution chart was obtained according to Formula 3.

## Results

### SE detection results

#### SE detection correction parameter calculation

**Figure 1:**
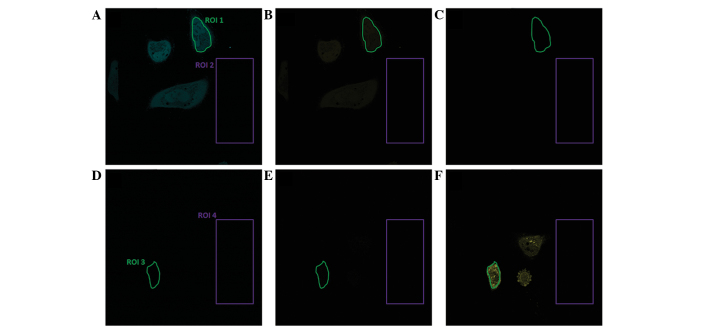
Image acquisition and parameter calculation in the sensitized emission (SE) method. (A–C) The samples transfected with cyan fluorescent protein (CFP)-particularly interesting new cysteine histidine-rich protein 1 (PINCH1) only. The correct focal plane and vision were selected to acquire the images in the three channels: (A) CFP, (B) FRET and (C) YFP. (D–F) The samples transfected with yellow fluorescent protein (YFP)-integrin-linked kinase (ILK) only. The correct focal plane and vision were selected to acquire the images in the three channels: (D) CFP, (E) FRET and (F) YFP. Regions of interest (ROIs) 1, 2, 3 and 4 were used to respectively select the fluorescence and background signals to calculate the values for α, β, γ and δ (data shown in Table I). FRET, fluorescence resonance energy transfer.

In the SE detection method, the images of the cell samples singly transfected with CFP-PINCH1 and YFP-ILK in the three channels (CPF, FRET and YFP) were initially acquired. The images of the singly transfected CFP-PINCH1 sample are shown in Fig. 1A–C. The images of the singly transfected YFP-ILK sample are shown in Fig. 1D–F. To select the ROI, the signal and background fluorescence values for calculating the net fluorescence value were counted. Formula 2 was used to calculate the α, γ, β and δ values (Table I).

#### SE method detection result

**Figure 2:**
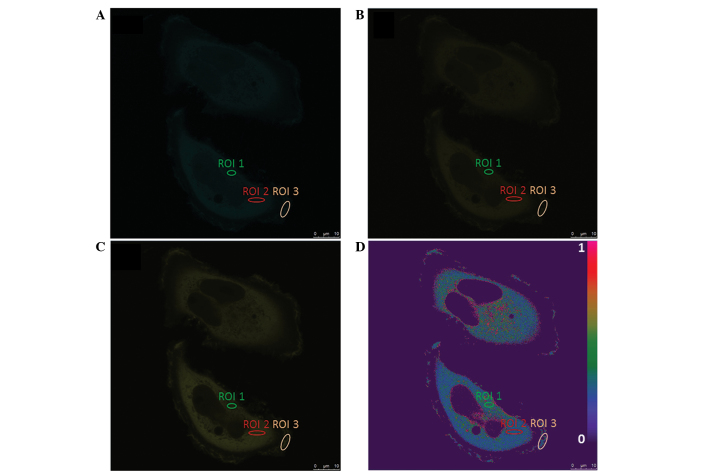
Fluorescence resonance energy transfer (FRET) efficiency distribution chart by the sensitized emission (SE) method. For the captured images of the (A) cyan fluorescent protein (CFP), (B) FRET and (C) yellow fluorescent protein (YFP) channels, computer software was used to calculate the FRET efficiency at each point in a point-to-point manner according to Form. 1. (D) The FRET efficiency distribution chart in full view was obtained, among which: region of interest (ROI) 1 refers to the cell tail, ROI 2 refers to the cytoplasm and ROI 3 refers to the leading edge.

For the captured images of the CFP, FRET and YFP channels (Fig. 2A–C), the Leica confocal software was used to calculate the FRET efficiency value of each point in a point-to-point manner according to Formula 2; thus, the FRET efficiency distribution chart in full view was obtained, as shown in Fig. 2D. The transition from purple to red demonstrates the increase in FRET efficiency from 0 (purple) to 1 (red), that is, the protein interaction from weak to strong.

The ROI was selected to calculate the FRET efficiency at different positions, as shown in Fig. 2D. The present study selected the ROIs according to the spatial differences between the PINCH1/ILK interactions, which included the cell tail (ROI 1), cytoplasm (ROI 2) and leading edge (ROI 3). The results of the calculations are shown in Table II. The FRET efficiency in the leading edge (ROI 3) was higher compared to that in the cell tail (ROI 1); however, the FRET efficiency in the cell tail (ROI 1) was higher compared to that in the cytoplasm (ROI 2).

#### AB method detection result

**Figure 3:**
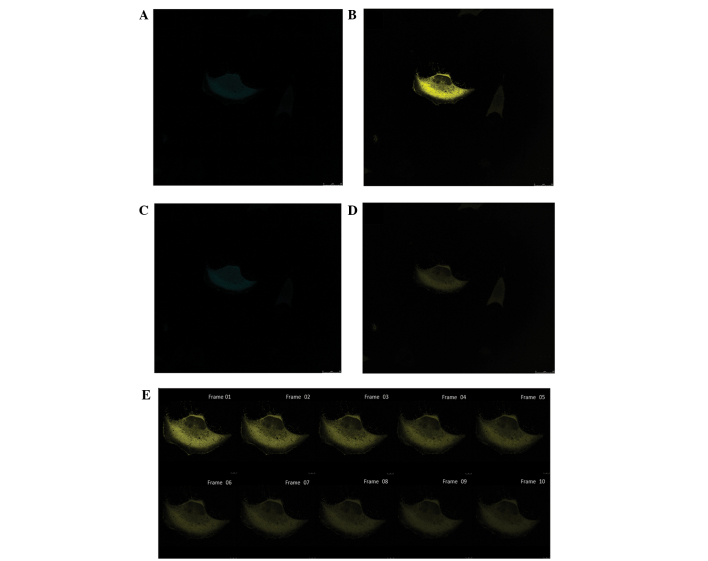
Image acquisition and acceptor fluorescent bleaching by the acceptor bleaching (AB) method. The region of interest (ROI) was selected and fluorescent bleaching was carried out on the receptor as many times as required. (A) The cyan fluorescent protein (CFP) fluorescence image prior to the acceptor fluorescent bleaching. (B) The yellow fluorescent protein (YFP) fluorescence image prior to fluorescent bleaching. (C) The CFP fluorescence image following fluorescent bleaching. (D) The YFP fluorescence image following fluorescent bleaching. (E) The process of fluorescent bleaching in the target area. ROI 1 refers to the cell tail, ROI 2 refers to the cytoplasm and ROI 3 refers to the leading edge.

Firstly, the cell images of the CFP and YFP channels were captured prior to acceptor fluorescent bleaching, as shown in Fig. 3A and B. Images were also acquired for the cells following bleaching, and representative images of the captured images in the CFP and YFP channels are shown in Fig. 3C and D. In the present experiment, the acceptor fluorescence was no longer reduced when the bleaching process had been undertaken 9 times. Therefore, the current study elected to carry out fluorescent bleaching 10 times, as shown in Fig. 3E.

**Figure 4:**
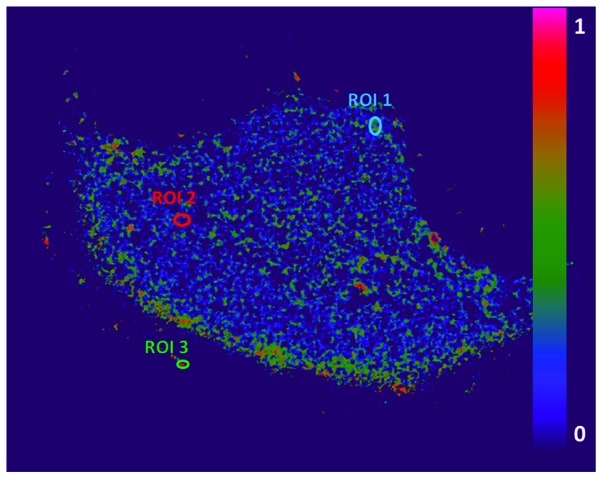
Fluorescence resonance energy transfer (FRET) efficiency distribution chart and cell movement affecting the detection result by the acceptor bleaching (AB) method. Computer software was used to calculate the FRET efficiency at each point in a point-to-point manner according to Formula 3; thus, the FRET efficiency distribution in full view was obtained. The region of interest (ROI) was selected to calculate the FRET efficiency of the target area (shown in Table III). ROI 1 refers to the cell tail, ROI 2 refers to the cytoplasm and ROI 3 refers to the leading edge.

According to the changes in donor fluorescence density in the cells prior to acceptor fluorescent bleaching, Formula 3 was used to obtain the statistical values and calculate the optical density of each point in the image, thereby obtaining the FRET efficiency distribution chart, as shown in Fig. 4. The transition from purple to red indicates the increase in the FRET efficiency from 0 (purple) to 1 (red), that is, it indicates the strength of the protein interaction from weak to strong. The ROI was selected to calculate the FRET efficiency at different positions. ROI 1 refers to the cell tail; ROI 2 refers to the cytoplasm and; ROI 3 refers to the leading edge, as shown in Fig. 4. The results of the calculations are shown in Table III. The FRET efficiency in the leading edge (ROI 3) was higher compared to that in the cell tail (ROI 1); however, the FRET efficiency in the cell tail (ROI1) was higher compared to that in the cytoplasm (ROI 2).

## Discussion

The use of FRET detection methods to investigate protein interactions in living cells has extensive prospects. However, there are various factors that affect the FRET detection results: i) the selection of FRET pairs, as the majority of the current FRET pairs exhibit the spectral crosstalk phenomenon. Thus, the selection of efficient FRET pairs is necessary (17). The R_0_, the effective overlapping of donor emission and acceptor excited spectroscopies and quantum yield are important factors for ensuring the high sensitivity of FRET detection (16). ii) The transfection effect. FRET detection methods require a high transfection effect of the target protein. iii) Donor and acceptor pairing. The full pairing of donors and acceptors seldom occurs in cells. Unpaired proteins interfere with signal detection. Furthermore, when the donor is paired with the acceptor, the dipole is required to have a certain spatial orientation. Otherwise, the FRET phenomenon does not occur even when the protein forms a compound (14). iv) Image acquisition. The quality of image acquisition and resolution also has an impact on the FRET detection result. In addition to the above influential factors, the selected detection method has a direct impact on the detection result.

SE has a low impact on fluorescence lifetime. SE is able to provide real-time detection of the protein interaction in the same sample for a long period of time. Thus, SE is favorable for dynamic studies of the interaction between proteins. The SE method is able to highly accurately detect the interaction between proteins with strong fluidity. In the AB method, only the donor signal is involved in the calculation of FRET efficiency. Therefore, no spectral crosstalk occurs. Furthermore, Figs. 2D and 4 show the leading edge. The occurrence range of FRET with more regular distribution detected by the SE method is wider compared with that detected by the AB method, indicating that the SE method is more sensitive than the AB method.

The AB method requires activated light to stimulate the samples numerous times over a long time period and has a serious impact on the optical activity of the acceptor fluorescent protein in the samples (as shown in Fig. 3E). Each sample may only be used once. The SE method is used to calculate the impact of the elimination of spectral crosstalk, background noise and other factors on FRET detection using strict parameter settings and formulas (19). The SE method does not have an impact on the optical activity of the fluorescent protein in the samples. The SE detection process is more complicated; however, the detection result is more accurate.

The SE detection result revealed that a spatial difference existed in the intensity of the PINCH1/ILK interaction. The PINCH1/ILK interaction in the leading edge was stronger compared with that in the cell tail; the PINCH1/ILK interaction in the cell tail was stronger compared with that in the cytoplasm (Table II). The AB detection result also demonstrated that the PINCH1/ILK interaction in the leading edge was stronger compared with that in the cell tail and the two were stronger compared with that in the cytoplasm (Table III). Thus, SE and AB were able to detect the intensity of the PINCH1/ILK interaction.

In conclusion, when FRET detection is performed on membrane proteins, the SE method is able to more sensitively and accurately detect the interaction between proteins in the cells. Thus, the SE method should be preferentially applied to the dynamic and real-time detection of protein interactions.

## Figures and Tables

**Table I:** Data acquisition results by the sensitized emission (SE) method.

	Transfected only by CFP-PINCH1 (donor, d)			Transfected only by YFP-ILK (acceptor, a)		
		
Channel	Signal value (ROI 1)	Background value (ROI 2)	Net value	Signal value (ROI 3)	Background value (ROI 4)	Net value
CFP (A)	61.94	2.77	59.17	2.91	2.90	0.01
FRET (B)	19.89	3.11	16.78	5.23	3.11	2.12
YFP (C)	3.08	3.07	0.01	78.31	3.10	75.21

CFP, cyan fluorescent protein; FRET, fluorescence resonance energy transfer; YFP, yellow fluorescent protein; ROI, region of interest; PINCH1, particularly interesting new cysteine histidine-rich protein 1; ILK, integrin-linked kinase. The following parameters were calculated from the net value data: α (A^a^/C^a^) = 0.000132961; β (B^d^/A^d^) = 0.283589657; γ (B^a^/C^a^) = 0.283589657; δ (A^a^/B^a^) = 0.004716981.

**Table II:** Calculation of FRET results by the sensitized emission (SE) method.

Channel	Cell tail (ROI 1)	Cytoplasm (ROI 2)	Leading edge (ROI 3)
CFP (A)	18.36	19.6	19.55
FRET (B)	16.72	14.06	20.5
YFP (C)	24.96	27.53	26.64
FRET efficiency (E)	0.409239491	0.32869058	0.502600446

CFP, cyan fluorescent protein; FRET, fluorescence resonance energy transfer; YFP, yellow fluorescent protein; ROI, region of interest.

**Table III:** Calculation of FRET efficiency by the acceptor bleaching (AB) method.

Characteristic	Cell tail (ROI 1)	Cytoplasm (ROI 2)	Leading edge (ROI 3)
Donor (prior to bleaching, I^b^_D_)	17.62	16.35	16.82
Donor (following bleaching, I^a^_D_)	22.00	19.34	44.20
Acceptor (prior to bleaching)	104.92	77.70	91.46
Acceptor (following bleaching)	37.49	22.80	41.05
FRET efficiency (E)	0.2568	0.1303	0.6014

FRET, fluorescence resonance energy transfer; ROI, region of interest.
